# Comparative evaluation of SUV, tumor-to-blood standard uptake ratio (SUR), and dual time point measurements for assessment of the metabolic uptake rate in FDG PET

**DOI:** 10.1186/s13550-016-0208-5

**Published:** 2016-06-22

**Authors:** Frank Hofheinz, Jörg van den Hoff, Ingo G. Steffen, Alexandr Lougovski, Kilian Ego, Holger Amthauer, Ivayla Apostolova

**Affiliations:** Helmholtz-Zentrum Dresden-Rossendorf, PET Center, Institute of Radiopharmaceutical Cancer Research, Bautzner Landstraße, Dresden, Germany; Department of Nuclear Medicine, University Hospital Carl Gustav Carus, Technische Universität, Dresden, Germany; Klinik für Radiologie und Nuklearmedizin, Universitätsklinikum Magdeburg A.ö.R., Magdeburg, Germany

**Keywords:** PET, FDG, Tumor-to-blood ratio, SUR

## Abstract

**Background:**

We have demonstrated recently that the tumor-to-blood standard uptake ratio (SUR) is superior to tumor standardized uptake value (SUV) as a surrogate of the metabolic uptake rate *K*_m_ of fluorodeoxyglucose (FDG), overcoming several of the known shortcomings of the SUV approach: excellent linear correlation of SUR and *K*_m_ from Patlak analysis was found using dynamic imaging of liver metastases. However, due to the perfectly standardized uptake period used for SUR determination and the comparatively short uptake period, these results are not automatically valid and applicable for clinical whole-body examinations in which the uptake periods (*T*) are distinctly longer and can vary considerably. Therefore, the aim of this work was to investigate the correlation between SUR derived from clinical static whole-body scans and *K*_m_-surrogate derived from dual time point (DTP) measurements.

**Methods:**

DTP ^18^F-FDG PET/CT was performed in 90 consecutive patients with histologically proven non-small cell lung cancer (NSCLC). In the PET images, the primary tumor was delineated with an adaptive threshold method. For determination of the blood SUV, an aorta region of interest (ROI) was delineated manually in the attenuation CT and transferred to the PET image. Blood SUV was computed as the mean value of the aorta ROI. SUR values were computed as ratio of tumor SUV and blood SUV. SUR values from the early time point of each DTP measurement were scan time corrected to 75 min postinjection (SUR_tc_). As surrogate of *K*_m_, we used the SUR(*T*) slope, *K*_slope_, derived from DTP measurements since it is proportional to the latter under the given circumstances. The correlation of SUV and SUR_tc_ with *K*_slope_ was investigated. The prognostic value of SUV, SUR_tc_, and *K*_slope_ for overall survival (OS) and progression-free survival (PFS) was investigated with univariate Cox regression in a homogeneous subgroup (*N*=31) treated with primary chemoradiation.

**Results:**

Correlation analysis revealed for both, SUV and SUR_tc_, a clear linear correlation with *K*_slope_ (*P*<0.001). Correlation SUR vs. *K*_slope_ was considerably stronger than correlation SUV vs. *K*_slope_ (*R*^2^=0.92 and *R*^2^=0.69, respectively, *P*<0.001). Univariate Cox regression revealed SUR_tc_ and *K*_slope_ as significant prognostic factors for PFS (hazard ratio (HR) =3.4/ *P*=0.017 and HR =4.3/ *P*=0.020, respectively). For SUV, no significant effect was found. None of the investigated parameters was prognostic for OS.

**Conclusions:**

Scan-time-corrected SUR is a significantly better surrogate of tumor FDG metabolism in clinical whole-body PET compared to SUV. The very high linear correlation of SUR and DTP-derived *K*_slope_ (which is proportional to actual *K*_m_) implies that for histologically proven malignant lesions, FDG-DTP does not provide added value in comparison to the SUR approach in NSCLC.

## Background

In a recent publication [1], we have demonstrated that the tumor-to-blood standard uptake ratio (SUR) is superior to tumor standardized uptake value (SUV) as a surrogate of the metabolic uptake rate *K*_m_ of [^18^F]fluorodeoxyglucose (FDG), overcoming several of the known shortcomings [2–6] of the SUV approach. In that work, we performed dynamic PET scans of liver metastases and computed lesion *K*_m_ using the Patlak method [7, 8] which was compared to lesion SUV and SUR in the late time frames. For these thus perfectly standardized uptake periods prior to SUV and SUR determination, we found that SUR correlated much better than SUV with the true metabolic rate of FDG.

However, in clinical oncological PET, variability of the uptake period is unavoidable [9, 10] which directly translates into a corresponding variability of the measured tracer uptake [11]. To avoid this limitation, a method to reliably correct SUR for variations of the FDG uptake period by converting SUR to a preselected fixed scan time point was proposed recently by our group [12]. This scan-time normalized SUR removes several of the shortcomings of SUV. We found strong evidence in a survival analysis of 130 patients with esophageal carcinoma that, consequently, SUR has a higher prognostic value than SUV [13]. However, these results were achieved in clinical whole-body scans with varying uptake periods. Thus, our previous results [1] regarding the correlation of SUR and *K*_m_ (with perfectly standardized and relatively short uptake period) are not necessarily valid for static oncological PET with variable uptake times. It remains an open question whether the improved prognostic value can actually be related to a linear correlation between SUR and *K*_m_ which is superior to that between SUV and *K*_m_.

Therefore, the aim of the present work was to investigate the correlation of SUV and SUR, respectively, with *K*_m_ in clinical whole-body scans. We evaluated 90 dual time point (DTP) measurements of patients with non-small cell lung cancer (NSCLC) which were used to derive the SUR slope *K*_slope_ as a surrogate parameter of *K*_m_ with a procedure similar to that in [14]. Secondary aim of this study was to test the prognostic value of SUV and SUR in comparison to *K*_slope_ in a homogeneous subgroup (*N*=31) of patients with NSCLC which underwent primary chemoradiation.

## Methods

### Assessment of metabolic uptake rate in dual time point measurement

In [14], we have shown that the metabolic uptake rate *K*_m_ of a tumor lesion can be estimated from a DTP measurement using two time points *T*_1_ and *T*_2_. There, we interpolated the arterial input function (AIF) *c*_*a*_(*t*) in the time window [*T*_1_,*T*_2_] using a single exponential. While mono-exponential interpolation is sufficient in the context of [14] and does not require knowledge or assumptions concerning the AIF outside the considered DTP time window, we have shown [12] that the shape of the AIF can be described globally (i.e., for all times after completion of the bolus passage) quite accurately by an inverse power law (i.e., a hyperbola)

1 $$  c_{a}(t) = A \times t^{-b}\quad\text{for}\quad t \gtrsim 1-2~\text{min} ~ \text{p.i}.  $$

where, moreover, *b* seems only modestly variable (≈± 10 *%*) across different investigations. (Precisely speaking, *t* in Eq. 1 has to be divided by the chosen time unit (e.g., 1 min.) to generate a dimensionless number that can be exponentiated; *A* then represents the AIF value at *t*=1 time unit.) Utilizing this information offers an alternative way of estimating *K*_m_ (or a proportional surrogate) from a DTP measurement. An immediate consequence of the hyperbolic AIF shape is that the so-called Patlak time

2 $$  \Theta(T) = \frac{{\int_{0}^{T}} c_{a}(t)dt}{c_{a}(T)}  $$

entering the Patlak equation [7, 8]

3 $$  \frac{c_{t}(T)}{c_{a}(T)} = K_{m}\times \Theta(T) + V_{r} \quad\text{for}\quad T \gtrsim 15-20~\text{min} ~ \mathrm{p.i}.  $$

(*c*_*t*_, tissue concentration; *V*_*r*_, apparent volume of distribution) is given by

4 $$  \Theta(T) = \frac{1}{1 - b} \times T\,.  $$

Recalling that SUR is by definition equal to the left-hand side of Eq. 3, the Patlak equation can be rewritten as

5 $$  \text{SUR}(T) = \frac{K_{m}}{1 - b} \times T + V_{r} = K_{\text{slope}} \times T + V_{r},  $$

In other words, if the AIF obeys Eq. 1 with some fixed value for *b*, SUR varies linearly with time for a given *K*_m_ and *V*_*r*_. Furthermore, *V*_*r*_ is numerically small and might be replaced by some group-averaged constant value $\bar {V}_{r}$ without introducing relevant errors (see [1]). SUR then also varies linearly with *K*_m_ (i.e., across different lesions/investigations/patients) at a given *T*. Equation 5 thus has two immediate consequence. The first one, as discussed in [12], is that scan time correction of SURs from actual measurement time point *T* to a reference time *T*_0_ is possible according to

6 $$  \text{SUR}(T_{0}) = \frac{T_{0}}{T} \times \left(\text{SUR}(T) - \bar{V}_{r} \right) + \bar{V}_{r}  $$

which allows to use scan-time-corrected SURs as a surrogate of *K*_m_. The second consequence is that *K*_m_ is directly related to the SUR slope,

7 $$  K_{m} = (1 - b) \times K_{\text{slope}},  $$

which can be derived from a DTP measurement as

8 $$  K_{\text{slope}} = \frac{\Delta \text{SUR}}{\Delta T} = \frac{\text{SUR}_{2} - \text{SUR}_{1}} {T_{2} - T_{1}}.  $$

If it is permissible to neglect inter- and intra-individual variability of the shape parameter *b* (i.e., assuming a generic AIF shape), we come to the conclusion that the DTP-derived SUR slope is directly proportional to *K*_m_ in an investigation-independent way. Of course, the above considerations rest on several assumptions whose validity cannot be taken for granted, especially if results from previous investigations are applied in a different context (e.g., different patient groups, much later scan times). Especially, notable deviations of the AIF from the assumed/extrapolated hyperbola at late times or violation of the assumed irreversible kinetics would affect the relation between instantaneous SUR at some fixed time point and the DTP-derived SUR slope.

In the present investigation, we therefore want to clarify the empirical relation between scan-time-corrected SUR (which is derivable from a standard whole-body investigation) and the SUR slope as derivable from DTP measurements and the extent to which this correlation is in agreement with the theory presented above according to which both quantities can serve as essentially equivalent surrogates of *K*_m_.

### Patient group

In the present study, 105 consecutive patients with histologically proven NSCLC were included retrospectively. Evaluation of the data was approved by the Institutional Ethics Committee, and all subjects provided written informed consent. In all patients, a routine dual time point FDG PET/CT was performed between March 2011 and June 2014 prior to treatment (radio(chemo)therapy (RCT) and/or surgery). Twelve patients were excluded because of a too short time difference between the two scans (*ΔT* <20 min) which compromises reliable determination of the DTP-derived SUR slope. Three patients were excluded because of misalignment of PET and attenuation CT affecting reliable SUR quantitation (see below). Altogether, 90 subjects were included (70 men, 20 females) with a mean age of (range) 67 (45–85) years. Validation of scan time correction of SUR and correlation analysis of *K*_slope_ vs. SUV and SUR, respectively, was performed in this group.

Survival analysis (see below) was performed in a homogeneous subgroup. Inclusion criteria were as follows: inoperable primary tumor, curative treatment intent, and no distant metastases. Altogether, 31 subjects were included in the survival analysis (27 men, 4 females) with a mean age of (range) 67 (49–85) years. Characteristics of the tumors are summarized in Table 1.

**Table 1:** Tumor characteristics

Characteristics	Number (%)
Histology	
Squamous cell carcinoma (SCC)	20 (65)
Adenocarcinoma (ADC)	11 (35)
T stage	
T1	5 (16)
T2	13 (42)
T3	6 (19)
T4	7 (23)
N stage	
N0	7 (23)
N1	3 (10)
N2	12 (39)
N3	9 (29)

### FDG PET/CT protocol

All patients underwent two hybrid FDG PET/CT. Scans (3D PET acquisition, 3 min per bed position) were performed with a Biograph mCT 64 (Siemens Medical Solutions Inc., Knoxville, TN, USA). Data acquisition of the early scan started (65.6±5.54) minutes (range 56.4–79.3) after injection of (234±9.02) MBq FDG. Acquisition of the late scans started (123±25.6) minutes postinjection (p.i.) (range 74.5–197). Time difference between the two respective scans was on average (52.9±23.8) minutes (range 20.4–131). All patients had fasted for at least 6 h prior to FDG injection. The serum glucose concentration measured prior to injection was 6.0 mmol/l on average (range 3.3–10.7). Furosemide (20 mg/2 ml) was injected intravenously 20 min after FDG application. Tomographic images were reconstructed using PSF + TOF reconstruction (2 iterations, 21 subsets). The resulting image data had a voxel size of 4.1×4.1×5 mm^3^.

### Image analysis

Coregistration, region of interest (ROI) definition, and ROI analyses were performed using the ROVER software, version 3.0.5 (ABX, Radeberg, Germany). Here and in the following, ROI is used synonymously with “VOI” for denoting a three-dimensional volume of interest.

For all PET data, the alignment with the attenuation CT (with focus on the tumor region) was inspected. Data were excluded when substantial parts of the FDG uptake was outside the morphological volume as measured in the CT data.

PET data of the late scan were coregistered to the PET data of the early scan using rigid body transformations. Coregistration was restricted to the tumor region plus a margin of 3–5 cm. Coregistration was visually inspected using the difference image of the late and early scans which allows to detect misalignments in the order of half of a voxel. Alignment was corrected manually when necessary. This was the case in 5 out of 90 cases.

**Fig. 1:**
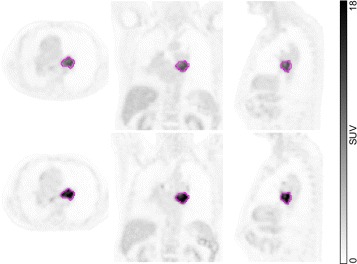
Representative orthogonal slices of a DTP measurement. Shown are the early scan (*top*) and the late scan (*bottom*). Both scans were coregistered with emphasis on the alignment of the primary tumor. The delineation of the tumor was performed in the early scan and the resulting ROI was transferred to the late scan. ROI boundaries are depicted in *magenta*

The metabolically active part of the primary tumor was delineated in the early scan by an automatic algorithm based on adaptive thresholding taking the local background into account [15, 16]. The result of the automatic delineation was inspected visually by an experienced observer and corrected manually in case of obvious segmentation failure. The resulting ROIs were transferred to the respective late scan (Fig. 1). In both scans, SUV_mean_ was computed. In the following, the index “mean” is omitted, since only the mean value of lesion SUV/SUR was considered in the evaluation. Lesion mean rather than maximum or peak (maximum + immediate vicinity) values where used since in the special case of DTP measurements, mean values can be determined with higher accuracy by performing precise coregistration and using identical delineation in both measurements. The usual problems typical of lesion mean values (systematic errors including partial volume effects due to variable delineation) thus affect both time points of the DTP measurement identically which minimizes their adverse effects. Maximum and peak values on the other hand do have distinctly higher statistical errors. Therefore, the minimum required time difference is distinctly larger when using maximum values which would not have been acceptable for the present retrospective evaluation of available data.

The arterial blood SUV was determined by defining a roughly cylindrical aorta ROI in the attenuation CT data which was then transferred to the PET data. To reduce partial volume effects, a concentric safety margin was used in the transaxial planes, centering the ROI in the aorta. Planes showing high tracer uptake close to the aorta (pathological or otherwise) were excluded. The minimum volume of the aorta ROI was 5 ml. Blood SUV was computed as mean SUV of the aorta ROI. The DTP pairs of blood SUVs were separately analyzed regarding consistency with the assumption of an invariant AIF shape (see Appendix).

SUR was computed as ratio of lesion SUV and blood SUV. *K*_slope_ was computed according to Eq. (8). SUR values from the early time point of each DTP measurement (SUR_1_) were scan time corrected to *T*_0_=75 min (SUR_tc_) using Eq. (6). The early time points were chosen since they correspond closely to the uptake times typical for static whole body investigations.

The performance of scan time correction was assessed by mean ± standard deviation (SD) of the fractional difference of SUR between late (SUR_2_) and early (SUR_1_) scan (*δSUR*=(SUR_2_−SUR_1_)/SUR_1_) before and after applying scan time correction to SUR_2_ from *T*_2_ to *T*_1_. Linear correlation analysis of SUV and, respectively, SUR_tc_ vs. *K*_slope_ was performed and visualized through scatterplots. Correlations were compared using a two-tailed *z*-test of the corresponding (Fisher transformed) correlation coefficients.

### Survival analysis

Survival analysis was performed in the patient subgroup described above (*N*=31). In this group, the association of the overall survival (OS) and progression-free survival (PFS) with SUV, SUR_tc_, and *K*_slope_ was analyzed using univariate Cox proportional hazard regression in which the PET parameters were included as binarized parameters. The cutoffs used for binarization were calculated by performing an univariate Cox regression for each measured value. The value leading to the hazard ratio (HR) with the highest significance was used as cutoff. The probability of survival was computed and rendered as Kaplan-Meier curves, and samples were compared using a log-rank test.

Statistical significance was assumed at a *P* value of less than 0.05. Statistical analysis was performed with the *R language and environment for statistical computing* [17] version 3.1.2.

## Results

**Fig. 2:**
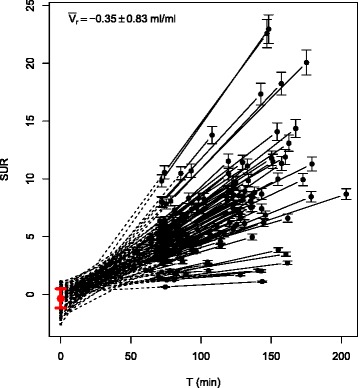
SURs as derived from the DTP measurements. *Black error bars* indicate estimated statistical errors derived by Gaussian error propagation when assuming a statistical accuracy of 5 % for blood SUVs and 2 % for the tumor SUVs. *Solid lines* connect the respective first and second time points of the DTP measurements. *Dashed lines* represent linear extrapolations back to *T*=0 yielding the individual *V*_*r*_ estimates for the respective DTP measurements. The *red point* and *error bars* are the mean and standard deviation of these *V*_*r*_ estimates. At the given level of accuracy, results are compatible with Eq. 5 according to which SUR(*T*) follows straight lines which approximately converge at a common point ($0,\bar {V}_{r}$)

Measured SURs are depicted in Fig. 2. The two respective DTP measurements (black circles) are connected with solid lines. The dashed lines represent linear extrapolations to *T*=0 yielding individual estimates of *V*_*r*_ whose average is $\bar {V}_{r} = (-0.35\pm 0.83)$ ml/ml. Consequently, the minor influence of a finite *V*_*r*_ might be neglected and $\bar {V}_{r}=0$ be used during scan time correction of SUR (Eq. 6) which thus reduces to SUR(*T*_0_)=*T*_0_/*T*×SUR(*T*).

**Fig. 3:**
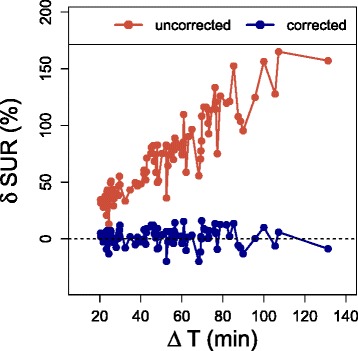
The fractional difference *δ*SUR between the first and second time points of each DTP measurement. *δ*SUR of the actual values (the fractional difference between measured SUR_2_ and SUR_1_ of the DTP pairs) is shown in *red*. *δ*SUR after scan time correction of SUR_2_ from *T*_2_ to *T*_1_ is depicted in *blue*

**Fig. 4:**
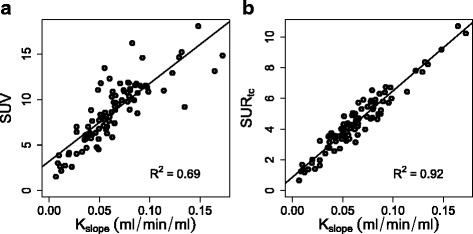
Correlation of SUV vs. *K*_slope_ (**a**) and of SUR_tc_ vs. *K*_slope_ (**b**)

*δ*Sur of the uncorrected values (i.e., the fractional difference between actually measured SUR_2_ and SUR_1_ of the DTP pairs) showed the expected strong dependency on *ΔT* with a large average value of (73±34) % (Fig. 3). After scan time correction of SUR_2_ from *T*_2_ to *T*_1_, the difference is essentially removed resulting in *δ*SUR=(1.5±7.6)*%*. Correlation analysis revealed for both, SUV and SUR_tc_, a clear linear correlation with *K*_slope_ (*P*<0.001). Correlation SUR_tc_ vs. *K*_slope_ was considerably stronger than correlation SUV vs. *K*_slope_(*R*^2^=0.92 and *R*^2^=0.69, respectively, *P*<0.001). While SUR_tc_ thus correlates highly with *K*_slope_ (and by implication with *K*_m_) (Fig. 4b), this is not the case for SUV (Fig. 4a) where large deviations from the regression line do occur.

**Fig. 5:**
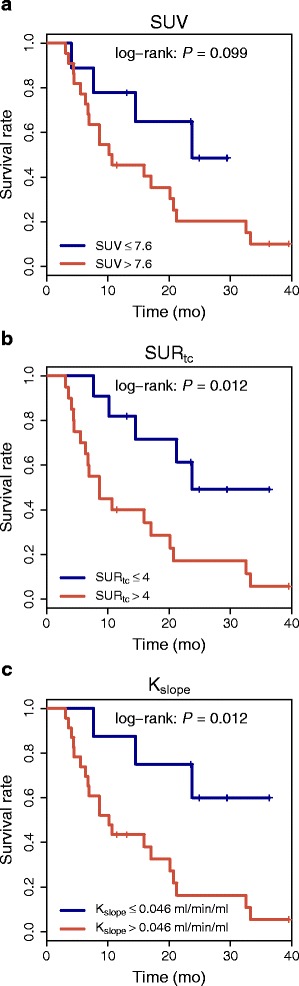
**a**–**c** Kaplan-Meier curves with respect to PFS

In survival analysis (*N*=31), univariate Cox regression revealed SUR_tc_ and *K*_slope_ as significant prognostic factors for PFS (HR =3.4/ *P*=0.017 and HR =4.3/ *P*=0.020, respectively). For SUV, no significant effect was found (Table 2). Corresponding Kaplan-Meier curves are shown in Fig. 5. None of the investigated parameters was prognostic for OS.

**Table 2:** Univariate Cox regression with respect to PFS

Parameter	Risk	HR	95 % CI	*P* value
SUV	>7.6	2.4	0.8–7.3	0.11
SUR_tc_	>4	3.4	1.2–9.3	0.017
*K*_slope_	>0.046 (ml/min/ml)	4.3	1.3–14.7	0.020

## Discussion

In this work, we investigated the correlation of SUV and scan-time-corrected SUR with the DTP-derived rate of SUR increase (SUR-slope), *K*_slope_. Accepting the results provided in the Appendix together with those of our previous studies [1, 12] as sufficient evidence for an essentially invariant hyperbolic AIF shape described by a unique value of the exponent *b* valid for the whole investigated patient group, *K*_slope_ is a proportional measure of *K*_m_. The main result of the current analysis is that SUR_tc_ correlates significantly better with *K*_slope_ (and thus *K*_m_) than is the case for SUV.

This finding is a direct consequence of the behavior apparent in Fig. 2 which demonstrates—in accordance with Eq. 5—that with good accuracy, all DTP-derived SUR pairs can be described by straight lines with quite small *y*-axis intercepts (parameter *V*_*r*_ in Eq. 5). While our previous results [1] yielded a mean of $\bar {V}_{r} \approx 0.53$ ml/ml, the data in Fig. 2 suggest rather to use $\bar {V}_{r} = 0$ ml/ml in the evaluation (which we did in the present paper). It should be emphasized that the precise choice for the (numerically small) value of $\bar {V}_{r}$ is of no major importance: using the previous best estimate $\bar {V}_{r} = 0.53$ ml/ml in the present investigation would just lead to a small bias of six percentage points in the computation of the scan-time-corrected *δ*SUR (blue points in Fig. 3) without any notable effect on the SUR_tc_ vs. *K*_slope_ correlation and the survival analysis. We further note that there is a visible negative correlation between SUR slope (and thus *K*_m_) and *V*_*r*_ in Fig. 2 (correlation coefficient *r*=−0.75), i.e., *V*_*r*_ tends to be smaller for larger *K*_m_, which also is to be expected on theoretical grounds. This correlation leads to a shift of the approximate point of convergence of the different straight lines. From a purely phenomenological point of view, this time shift might be accounted for in the equation relating SUR_tc_ and *K*_slope_ (while adjusting $\bar {V}_{r}$ accordingly) but we prefer to avoid this ad hoc approach and instead just use the $\bar {V}_{r} = 0$ approximation. This is perfectly adequate as demonstrated by our results.

Regarding the SUR_tc_ vs. *K*_m_ correlation, the current results are of comparable quality to that observed in [1] (*R*^2^=0.92 compared to *R*^2^=0.96), where the correlation of SUR and *K*_m_ as derived from Patlak analysis of dynamic studies up to 60 min p.i. was investigated (see Figure 4 in [1]). Our results are also in accord with previous findings by Hunter et al. which used a somewhat different but ultimately mostly equivalent approach [18] as well as with a recent study by Grecchi et al. [19] which demonstrates the much improved correlation of SUR—referred to as “ratio method” in that paper—with *K*_m_ compared to that of SUV vs. *K*_m_ in a different context, namely patients with acute lung injury. The present study further supports the observation that SUR_tc_ is a better surrogate of tumor FDG metabolism than SUV. In the current analysis, we especially demonstrated validity of this assumption for later times points p.i. (and in a different tumor entity).

This finding is also in accord with the performed survival analysis. While there was no significant effect for SUV, both SUR_tc_ and *K*_slope_ were significant prognostic factors for PFS with comparable effect size. Since it is clear that the sample size available for this analysis (*N*=31) is far too small for conclusive results, further investigations will be necessary to clarify this point. Nevertheless, our results still are an indication that the increased correlation of SUR_tc_ with *K*_slope_ compared to SUV translates into an increased prognostic value. Since SUR_tc_ and *K*_slope_ showed almost the same prognostic value, it can be stated that for histologically proven NSCLC DTP measurements seem not to provide additional information compared to SUR_tc_ analysis of static whole-body scans. We presume that this conclusion is generally valid as long as no large deviations from irreversible FDG kinetics are to be expected.

Our analysis rests on the assumption that the AIF can be described by a hyperbola (Eq. 1). Therefore, the results regarding scan time correction (Fig. 3) are of special interest. Scan time correction will work correctly only if the AIF can be described by a power law. Our results thus strongly support this assumption. To some extent, this was already shown in [12]. Here, we were able to confirm these results in a larger patient group and for a larger range of uptake periods.

As has already been explained, *K*_slope_ is a proportional substitute of the actual *K*_m_ across different investigations and patients if the AIF follows Eq. 1 with a unique value of the exponent *b*, i.e., the shape need not only be hyperbolic but also be invariant across investigations. The actual numerical value taken on by *b* is irrelevant as long as one is not interested in quantitatively deriving *K*_m_ from *K*_slope_ (or SUR_tc_). It is, however, relevant to ensure that shape invariance of the AIF with a constant *b* is a valid assumption in order to compare *K*_slope_ or SUR_tc_ from different investigations. Figures 2 and 4b do not allow any direct conclusions in this respect (and neither does Fig. 3). But the fact that, indeed, *K*_slope_ as well as SUR_tc_ perform superior to SUV in the survival analysis supports the conjecture that the former two quantities are in fact better correlated with *K*_m_ which in turn implies that *b*≈const in the whole patient group. The detailed analysis of the blood SUV data presented in the Appendix supports the hypothesis that the AIF actually adheres to an invariant shape.

## Conclusions

Scan-time-corrected SUR is a significantly better surrogate of tumor FDG metabolism in clinical whole-body PET compared to SUV. The very high linear correlation of SUR and DTP-derived *K*_slope_ (proportional to the actual *K*_m_) implies that for histologically proven malignant lesions, FDG-DTP does not provide added value in comparison to the SUR approach in NSCLC. The potential benefit of DTP for differentiation of malignant and inflammatory or benign lesions with high uptake should be established in further studies.

## Compliance with ethical standards

All procedures performed in studies involving human participants were in accordance with the ethical standards of the institutional and/or national research committee and with the 1964 Helsinki declaration and its later amendments or comparable ethical standards. Informed consent was obtained from all individual participants included in the study.

## Appendix

The results presented in Fig. 4b, demonstrate a very good linear correlation of SUR_tc_ with *K*_slope_ in full accord with the prediction of Eq. 5. Ultimately, this implies that the AIF is adequately parameterized by the hyperbola Eq. 1 (scan time correction to some reference time would not work otherwise while being a prerequisite of considering Eq. 5 for a fixed time *T* and predicting the correlation Fig. 4b). On the other hand, these results do not allow any direct conclusion regarding a generic (investigation-independent constant) value of the power law exponent *b* since only the ratio *K*_slope_=*K*_m_/(1−*b*) appears in Eq. 5 and *K*_m_ is not known a priori. But only if *b* can be assumed to be constant (and thus the AIF to be shape-invariant) across different investigations, *K*_slope_ (and SUR_tc_) are valid surrogates of *K*_m_ when comparing data from different patients/investigations. Only the fact that *K*_slope_ as well as SUR_tc_ indeed perform superior to SUV in the survival analysis (and thus should be more closely related to *K*_m_ and, ultimately, to glucose consumption) provides indirect evidence for *b*≈const.

**Fig. 6:**
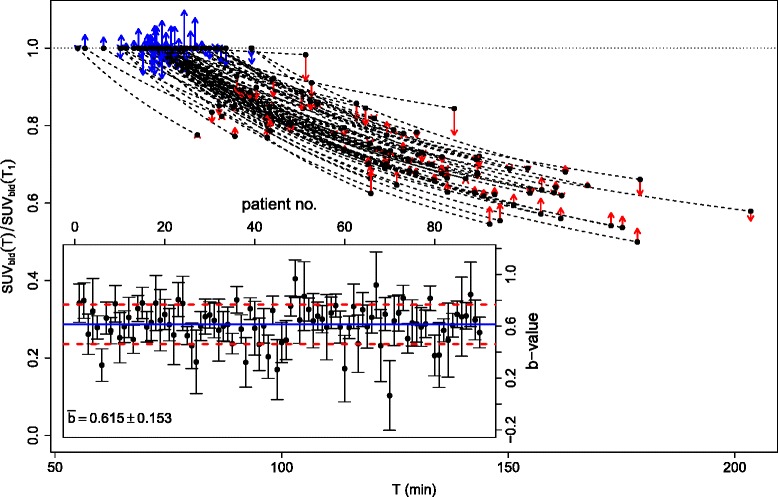
Pairs of blood SUVs for all DTP measurements. Shown are the ratios of the second to the first value. *Dashed lines* are the hyperbolas connecting the pairs. The corresponding *b* values are shown in the *inset*. For further details, see Appendix

The purpose of the present Appendix is to provide direct evidence that the *b*≈const assumption is valid for our patient group. For this purpose, Fig. 6 shows the relative decrease of blood SUV from the first to second measurements for all DTP pairs. The dashed lines connecting both points are the hyperbolas according to Eq. 1. Blue/red arrows indicate the deviation of the first/second measurements from the mean hyperbola (corresponding to the group-averaged *b* value).

The inset shows the individual *b* values for all DTP pairs as well as their mean (blue line) and standard deviation (dashed red lines). Individual error bars are determined by Gaussian error propagation using a realistic estimate of 5 % for the statistical accuracy of the separate blood SUV measurements.

As can be seen, the deviation of the individual blood SUVs from the mean hyperbola is quite small. The inset, furthermore, demonstrates, that the standard deviation of the *b* value distribution compares favorably with the error bars of the individual *b* values. Altogether, the data thus do not provide any evidence at the given level of measurement accuracy that the individual *b* values are significantly different, i.e., the assumption of a common AIF shape (at least in the accessible time window) is fulfilled in this patient group. As already explained, this suffices to ensure that *K*_slope_ as well as SUR_tc_ can act as accurate surrogates of *K*_m_ which in our view is the underlying explanation for superior performance of both parameters in comparison to SUV in the survival analysis.

Obviously, knowledge of the actual numerical value of *b* is irrelevant (as long as it remains constant across investigations) if one is not interested in quantitatively deriving *K*_m_ from *K*_slope_ or SUR_tc_. While the actual numerical value of *b* thus is of no importance for the present paper, it seems necessary to point out that the average *b* value derived from the data in Fig. 6 ($\bar {b} = 0.615 \pm 0.153$) is distinctly larger—corresponding to a more pronounced decrease of the AIF over time—than our previously published value $\bar {b} = 0.313 \pm 0.030$). While the latter value is more reliable (being based on full, dynamic AIFs) the underlying data were restricted to times ≤60 min, whereas the present data cover distinctly later times after injection. For the present data, the substantial discrepancy between both *b* values still only implies on average a ≈±8 *%* change of the first/second blood SUV of the DTP measurement in comparison to what would have been expected from the previous results. Presently, it cannot be ruled out completely that this effect is real (rather than some unidentified systematic small error in the data such as contrast-dependence of the scanner’s image reconstruction software) and that the AIF shape is deviating from simple hyperbolic behavior beyond 60 min p.i. Based on data from an independent ongoing investigation, we think this to be improbable but this question deserves further attention.
